# Role of caregiver factors in outpatient medical follow-up post-stroke: observational study in Singapore

**DOI:** 10.1186/s12875-021-01405-z

**Published:** 2021-04-14

**Authors:** Shilpa Tyagi, Gerald Choon-Huat Koh, Nan Luo, Kelvin Bryan Tan, Helen Hoenig, David B. Matchar, Joanne Yoong, Angelique Chan, Kim En Lee, N. Venketasubramanian, Edward Menon, Kin Ming Chan, Deidre Anne De Silva, Philip Yap, Boon Yeow Tan, Effie Chew, Sherry H. Young, Yee Sien Ng, Tian Ming Tu, Yan Hoon Ang, Keng He Kong, Rajinder Singh, Reshma A. Merchant, Hui Meng Chang, Tseng Tsai Yeo, Chou Ning, Angela Cheong, Yu Li Ng, Chuen Seng Tan

**Affiliations:** 1 Saw Swee Hock School of Public Health, National University of Singapore, 12 Science Drive 2, #10-01, Singapore, 117549 Singapore; 2 Policy Research & Economics Office, Ministry of Health, Singapore, Singapore; 3 Physical Medicine and Rehabilitation Service, Durham VA Medical Centre, Durham, NC USA; 4 Program in Health Services and Systems Research, Duke-NUS Medical School, Singapore, Singapore; 5 Lee Kim En Neurology Pte Ltd, Singapore, Singapore; 6 Raffles Neuroscience Centre, Raffles Hospital, Singapore, Singapore; 7 St. Andrew’s Community Hospital, Singapore, Singapore; 8 Mount Alvernia Hospital, Singapore, Singapore; 9 National Neuroscience Institute, Singapore General Hospital Campus, Singapore, Singapore; 10 Geriatric Medicine, Khoo Teck Puat Hospital, Singapore, Singapore; 11 St. Luke’s Hospital, Singapore, Singapore; 12 Department of Rehabilitation Medicine, National University Hospital, Singapore, Singapore; 13 Department of Rehabilitation Medicine, Changi General Hospital, Singapore, Singapore; 14 Department of Rehabilitation Medicine, Singapore General Hospital, Singapore, Singapore; 15 Department of Neurology, National Neuroscience Institute, Neurology, Tan Tock Seng Hospital, Singapore, Singapore; 16 Department of Rehabilitation Medicine, Tan Tock Seng Hospital, Singapore, Singapore; 17 Department of Medicine, Yong Loo Lin School of Medicine, National University of Singapore, Singapore, Singapore; 18 Department of Neurosurgery, National University Hospital, Singapore, Singapore

**Keywords:** Primary care, Caregivers, Stroke, Healthcare utilization, Family caregivers

## Abstract

**Background:**

Outpatient medical follow-up post-stroke is not only crucial for secondary prevention but is also associated with a reduced risk of rehospitalization. However, being voluntary and non-urgent, it is potentially determined by both healthcare needs and the socio-demographic context of stroke survivor-caregiver dyads. Therefore, we aimed to examine the role of caregiver factors in outpatient medical follow-up (primary care (PC) and specialist outpatient care (SOC)) post-stroke.

**Method:**

Stroke survivors and caregivers from the Singapore Stroke Study, a prospective, yearlong, observational study, contributed to the study sample. Participants were interviewed 3-monthly for data collection. Counts of PC and SOC visits were extracted from the National Claims Database. Poisson modelling was used to explore the association of caregiver (and patient) factors with PC/SOC visits over 0–3 months (early) and 4–12 months (late) post-stroke.

**Results:**

For the current analysis, 256 stroke survivors and caregivers were included. While caregiver-reported memory problems of a stroke survivor (IRR: 0.954; 95% CI: 0.919, 0.990) and caregiver burden (IRR: 0.976; 95% CI: 0.959, 0.993) were significantly associated with lower early post-stroke PC visits, co-residing caregiver (IRR: 1.576; 95% CI: 1.040, 2.389) and negative care management strategies (IRR: 1.033; 95% CI: 1.005, 1.061) were significantly associated with higher late post-stroke SOC visits.

**Conclusion:**

We demonstrated that the association of caregiver factors with outpatient medical follow-up varied by the type of service (i.e., PC versus SOC) and temporally. Our results support family-centred care provision by family physicians viewing caregivers not only as facilitators of care in the community but also as active members of the care team and as clients requiring care and regular assessments.

**Supplementary Information:**

The online version contains supplementary material available at 10.1186/s12875-021-01405-z.

## Background

Stroke presents a major public health challenge accounting annually for 16 million cases and 5.7 million deaths globally [1].Stroke is the third largest contributor of DALYs [2], the tenth most common cause of hospitalization [3] and one of the top five causes of death in Singapore [4]. Treatment of stroke comprises of an acute and sub-acute or early chronic phase. While the acute phase includes emergency care in a tertiary inpatient setting, the sub-acute or early chronic phase comprises rehabilitation and outpatient medical follow-up to ensure continuity of care and implement secondary prevention practices. The outpatient medical follow-up usually occurs in a primary care (PC) setting involving a family physician. Researchers in a study reported family physicians (93.2%) to be the most frequently visited healthcare professional post-stroke, followed by other medical specialists (54%) [5].

Being voluntary and non-urgent, outpatient medical follow-up in PC or specialist outpatient care (SOC) setting is potentially determined by both healthcare needs and overall socio-demographic context of caregivers and stroke survivors. Moreover, stroke survivors often have residual impairments of varying magnitude, which make it challenging to attend outpatient medical visits independently. Often a family member engaged in caregiving responsibilities assists with such healthcare tasks [6] highlighting the relevance of including caregivers in studies on outpatient medical follow-up post-stroke. Evidence suggests that caregivers’ involvement in care plans of their care recipients and providing adequate education to the caregivers can decrease costs and increase patient satisfaction [7].

A systematic review involving 168 stroke survivors and 328 informal caregivers synthesized findings from 51 qualitative studies exploring primary care and community services experiences. They reported stroke survivor-caregiver dyads feeling marginalized by inadequate information provision, limited continuity of care and access to services post-stroke. None of the studies were from an Asian setting and the scope of the review excluded quantitative studies, which may provide more generalizable findings [8]. A cross-sectional study in Canada comparing healthcare visits of patients with and without stroke reported patients with stroke were twice more likely to visit a medical specialist and 1.5 times more likely to visit a family physician as compared to patients without stroke. Although researchers explored the association of outpatient medical healthcare visits with stroke survivors’ demographics, mobility, comorbid cardiometabolic conditions, and mood/anxiety disorders, they did not examine the association with any caregiver factor in their study [5]. Roth and colleagues used Medicare claims data to study health service utilization over six months after index stroke hospitalization in 279 stroke survivors from the REGARDS (REasons for Geographic And Racial Differences in Stroke) study in the US. They reported that co-residing with a caregiver was associated with reduced health service utilization, including shorter hospital lengths of stay, fewer emergency department visits, and fewer primary care visits [9]. Another study in the US exploring the contribution of caregiver factors in healthcare service utilization by stroke survivors reported caregiver’s health beliefs being associated with stroke survivors attending scheduled medical and therapy appointments post-discharge from inpatient rehabilitation facilities [10]. Among these existing studies exploring the role of caregivers in outpatient medical follow-up, most of the studies are either qualitative [8] or conducted in Western settings [5, 9, 10] and had limited [9, 10] or no inclusion of caregiver factors [5]. Addressing the existing gaps, we aimed to examine the association of caregiver factors (along with patient factors) with outpatient medical follow-up of stroke survivors over 1-year post-stroke. The outpatient medical follow-up comprised of PC and SOC visits. PC visits consisted of stroke survivor’s visits to any public PC clinics in Singapore during the yearlong follow-up. Also known as “*one stop primary care clinics*,” public PC clinics are government run, providing subsidized care to Singaporeans and permanent residents. They are multi-doctor led clinics, which provide a comprehensive range of services [11]. While the Singapore healthcare system consists of a mixed public–private primary care, almost half (45%) of patients with chronic diseases are managed in public PC clinics [12]. SOC visits included attendance to any of the specialist run outpatient clinics in the tertiary hospital premises.

## Methods

We conducted a yearlong, prospective, observational study involving stroke survivors and their caregivers recruited from all five tertiary hospitals in Singapore during the recruitment period, from December 2010 to September 2013. The eligibility criteria for stroke survivors included the following: (a) Singaporean or permanent resident, more than 40 years old and residing in Singapore for the next one year, (b) stroke must be a recent diagnosis (i.e., stroke symptoms occurring within four weeks before admission) with the diagnosis made by a clinician and/or supported by brain imaging (CT or MRI) and (c) not globally aphasic. The eligibility criteria for the caregivers included the following: (a) an immediate or extended family member or friend, (b) more than 21 years of age (the legal definition of adult in Singapore), (c) providing care or assistance of any kind and taking responsibility for the patient (as recognized by the patient) and (d) not fully paid for caregiving. We did not impose any language related exclusions, enabling us to recruit a multi-ethnic study cohort. The study procedures are reported in detail separately [13, 14]. This study was approved by the National University of Singapore Institutional Review Board, SingHealth Centralized Institutional Review Board, and the National Health Group Domain Specific Review Board. Written informed consent was obtained from both the patients and the caregivers in their preferred language by trained researchers. All methods were performed in accordance with the guidance provided in the Declaration of Helsinki.

### Data collection

We administered face-to-face verbal questionnaires to both stroke survivors and caregivers at baseline, 3-month and 12-month time points. Only caregivers were administered verbal questionnaires via telephone at 6-month and 9-month time points. The data collected in these surveys were broadly categorized under the health, social, and financial domains, each comprising patient, caregiver, and self-reported healthcare utilization variables, respectively [14, 15]. The main difference between face-to-face and telephone surveys was capturing all three domains during face-to-face surveys, while only financial domain data was captured during telephone surveys. We extracted the dependent variables from the National Claims Database, which has been reported to be a more objective source of healthcare data [16]. The National Claims Database is a nation-wide database of healthcare utilization and associated expenditure maintained centrally by the Ministry of Health in Singapore. With the aid of a unique identification number allocated to all Singapore citizens and permanent residents, we linked our prospective cohort data with the healthcare data in the National Claims Database achieving a match rate of more than 95%. The independent variables were taken from the baseline and 3-month face-to-face verbal questionnaires of our observational study (i.e., patient variables at baseline and caregiver variables at 3-month).

### Dependent variables

The primary outcome variable was counts of PC visits and SOC visits within the first three months post-stroke (i.e., early post-stroke period or 0–3 months). In addition, we separately examined PC and SOC visits 4–12 months post-stroke or late post-stroke period. PC visits consisted of stroke survivor’s visits to any public PC clinics in Singapore during the yearlong follow-up. For current analysis, we did not include home visiting services by family physicians or general practitioners.

### Independent variables

Our primary independent variables were caregiver factors assessed at 3 months post index stroke admission: socio-demographic variables, caregiver relationship, comorbid conditions, co-residing with the care recipient (i.e., whether the caregiver lived with the stroke survivor in the same house), caregiver-reported patient behavioral problems, caregiver burden, family conflict, social support and caregiver-adopted care management strategies. We used the revised dementia management strategies scale to capture caregiver-adopted care management strategies. The 20-item instrument version has been validated in Singapore [17] and records responses to the frequency of adopted strategy on a 5-point Likert scale: 1 = never, 2 = seldom, 3 = sometimes, 4 = often and 5 = most of the time. The instrument comprises two subscales of positive and negative dimensions, with good reported internal consistency in the Singapore population (Cronbach’s alpha 0.89 and 0.87 respectively) [17]. We summated the total score across these two dimensions of positive and negative care management strategies. An example of a statement under positive care management strategy was, “I made it a point to praise him when he did what I considered appropriate.” An example of a statement under the negative care management strategy was, “I yelled or acted angry, it was often the only way to get my way with him.”

We considered the following patient variables collected at baseline: socio-demographic variables, marital status, ward class, comorbid condition (captured using Charlson Comorbidity Index, CCI) [18], stroke type (ischemic or non-ischemic including hemorrhagic and mixed); recurrent stroke, stroke severity (measured using National Institute of Health Scale, NIHSS) [19], level of disability (measured using Modified Rankin Scale, mRS) [20], cognitive impairment (using the Mini-Mental State Examination, MMSE) [21] and discharge destination. For scales with significant (> 10) number of missing cases (NIHSS, MMSE, Revised memory and behavior checklist), we used the person mean substitution approach to impute for missing values for cases with less than half the constituent items missing [22]. Please refer to Additional file 1 for detailed description of independent variables included in the study.

### Data analysis

Univariate analysis was performed to describe our sample of stroke patient-caregiver dyads. Bivariate analysis was performed using Poisson modelling to examine the associations between caregiver/patient factors and the risk of PC/SOC visits post-stroke. Independent variables (caregiver and patient factors) having *p*-value < 0.1 in the bivariate analyses were chosen as potential predictors for the multivariable Poisson regression model. Using these potential predictors, a backward variable selection approach was conducted to identify the most parsimonious model by removing the least significant variable at each model building iteration step with a p-value for inclusion < 0.05 (except for age, gender, ethnicity and ward class of the patient with stroke which we opted to retain in the model a priori to standardize for socio-demographic variables). With the most parsimonious adjusted model, we assessed for over-dispersion and excessive zeroes using alpha statistic and Voung test, respectively [23, 24] and applied the appropriate regression model accordingly. We reported the unadjusted and adjusted incidence rate ratio (IRR) estimates with 95% confidence intervals (CI). Separate models were run for PC visits and SOC visits for the following two time periods: first 3 months post-stroke and subsequent 4–12 months post-stroke. Results are reported based on a significance level set at 5%. All analysis was performed in Stata version 14.1 [25].

## Results

**Fig. 1:**
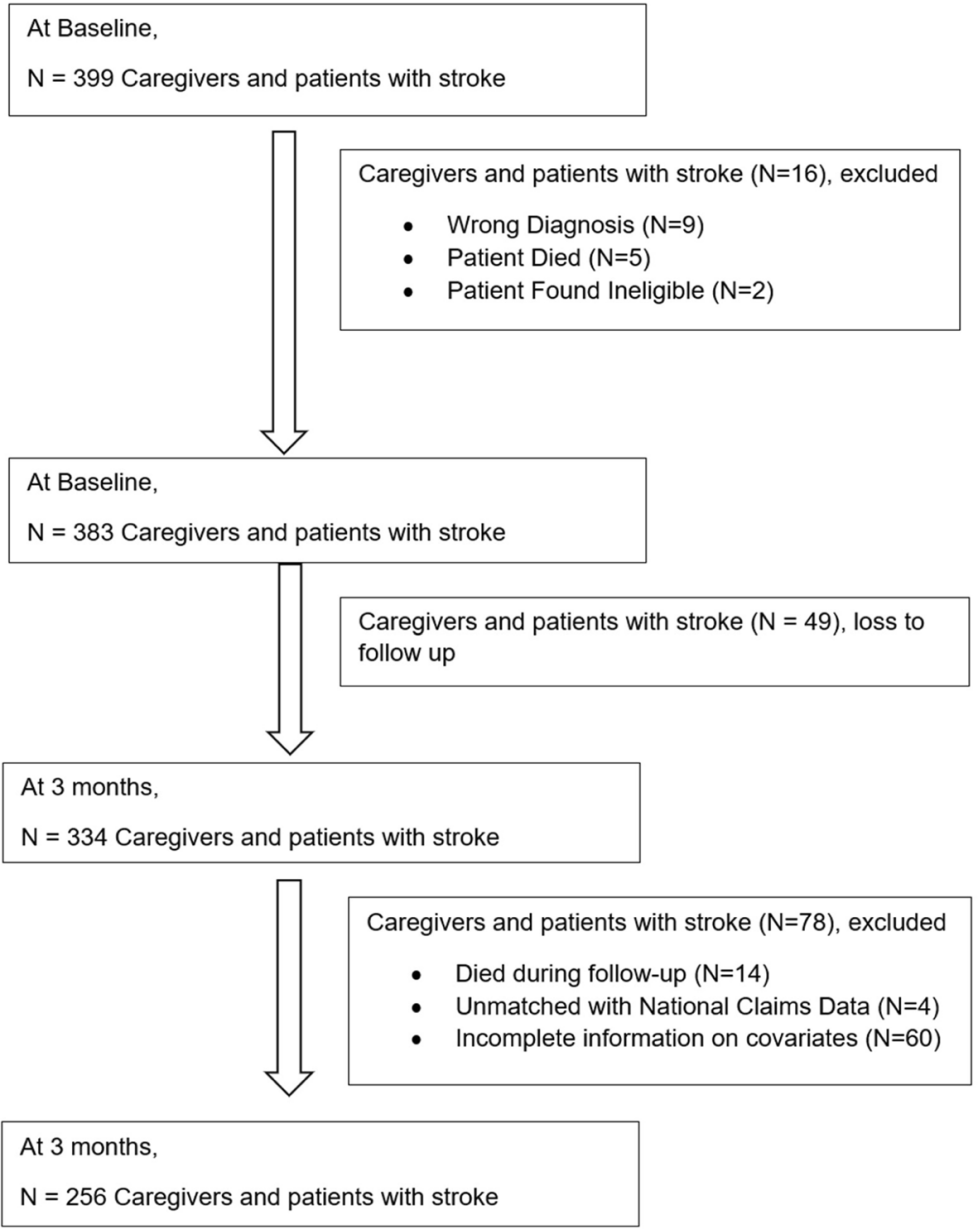
Study flowchart

Two hundred and fifty-six stroke survivor and caregiver dyads were available for the current analysis. (please refer to Fig. 1). The average (standard deviation or SD) number of PC visits for stroke survivors over 0–3 months and 4–12 months post-stroke were 0.867 (1.197) and 2.305 (2.242), respectively. The average (SD) number of SOC visits for stroke survivors over 0–3 months and 4–12 months post-stroke were 1.836 (1.823) and 4.605 (5.616), respectively. The average age of caregivers was 50 years, with 76% female, 57% Chinese and 79% married. With the mean age of 61.8 years, the majority of the stroke survivors were male (64%), Chinese (58%) and married (80%). Eighty-nine percent of the stroke survivors had an ischemic stroke, with 25% discharged to a step-down facility from an acute hospital setting (please refer to Table 1).

**Table 1:** Descriptive characteristics of caregivers and patients

	No. (%) unless otherwise stated	
**Caregiver**^**a**^**factors**		
**Age (in years)**	Mean (SD)	50.0 (13.2)
**Gender**	Male	61 (24)
	Female	195 (76)
**Ethnicity**	Chinese	147 (57)
	Non-Chinese	109 (43)
**Marital status**	Married	201(79)
	Single	55 (21)
**Caregiver relationship**	Spouse	155 (60)
	Adult-Child	74 (29)
	Sibling	10 (4)
	Others	17 (7)
**Comorbid conditions**	None	153 (60)
	1	57 (22)
	2	24 (9)
	≥3	22 (9)
**Co-residing with patient**	Yes	227 (89)
	No	29 (11)
**Caring for multiple care recipients**	Yes	106 (41)
	No	150 (59)
**Revised memory and behavior checklist**		
**Memory problems**	Mean (SD)	5.1 (6.0)
**Depressive behavior problems**	Mean (SD)	3.2 (4.9)
**Disruptive behavior problems**	Mean (SD)	2.7 (3.6)
**Caregiver burden**		
**Oberst caregiving burden scale**	Mean (SD)	31.8 (12.7)
**Zarit burden interview**	Mean (SD)	8.8 (7.9)
**Family conflict**		
**Attitude towards patient**	Mean (SD)	11.4 (4.5)
**Attitude towards caregiver**	Mean (SD)	11.6 (4.4)
**Social support (instrumental)**		
**FDW for general help**	Yes	208 (81)
	No	48 (19)
**FDW for stroke patient**	Yes	33 (13)
	No	223 (87)
**Social support (perceived)**	Mean (SD)	26.4 (4.9)
**Care management strategies**		
**Positive strategies**	Mean (SD)	34.5 (10.8)
**Negative strategies**	Mean (SD)	11.1 (4.6)
**Patient**^**b**^**factors**		
**Age (in years)**	Mean (SD)	61.8 (10.5)
**Gender**	Male	165 (64)
	Female	91(36)
**Ethnicity**	Chinese	149 (58)
	Non-Chinese	107 (42)
**Marital status**	Married	206 (80)
	Single	50 (20)
**Ward class**	Unsubsidized	21 (8)
	Subsidized	235 (92)
**Charlson Comorbidity Index (during stroke onset)**	1 - 3	52 (20)
	4 - 6	162 (64)
	≥ 7	42 (16)
**Stroke type**	Ischemic	227 (89)
	Non-ischemic	29 (11)
**Recurrent stroke**	Yes	42 (16)
	No	214 (84)
**National Institute of Health Scale**	Mild (0-4)	145 (57)
	Moderately severe (5-14)	97 (38)
	Severe (15-24)	14 (5)
**Modified rankin scale**	No or slight disability (0-2)	105 (41)
	Moderate or severe disability (3-5)	151 (59)
**Mini-mental state examination**	No (24-30)	147 (57)
	Mild (18-23)	64 (25)
	Severe (1-17)	45 (18)
**Discharge to community hospital or step-down facility**	Yes	65 (25)
	No	191 (75)
**Centre for epidemiological studies depression scale**	Mean (SD)	6.3 (5.6)

Source: Tyagi S, Koh GCH, Luo N, Tan KB, Hoenig H, Matchar DB, et al. Dyadic approach to post-stroke hospitalizations: role of caregiver and patient characteristics. BMC Neurology. 2019;19(1):267

*Abbreviations*: *No.* number, *SD* standard deviation, *FDW* foreign domestic worker

^a^Variables collected at 3-month time point

^b^Variables collected at baseline

### Outpatient medical (PC) healthcare utilization

Table 2 depicts the results of the association of caregiver and patient characteristics with the rate of PC visits across three months post-stroke. The variables that entered the final adjusted model of PC visits were caregiver-reported patient behavioral problems (memory), objective caregiver burden measured on the Oberst caregiving burden scale and stroke type. For every 1 unit increase in caregiver-reported patient memory problem score, the expected rate ratio of PC visits decreased by a factor of 0.954 (95% CI: 0.919, 0.990). With every 1 unit increase in the Oberst caregiver burden scale score, the rate ratio of PC visits decreased by a factor of 0.976 (95% CI: 0.959, 0.993). The variables that entered the final adjusted model of PC visits over 4–12 months were only patient factors, specifically, CCI, stroke type and stroke severity. (Please refer to Table 3) Compared to stroke survivors with CCI score of 1–3, the rate ratio of PC visits over 4–12 months post-stroke was 1.655 (95% CI: 1.152, 2.378) times higher in those with CCI score of 4–6. Compared to those with non-ischemic stroke, the rate ratio of PC visits over 4–12 months post-stroke was 1.867 (95% CI:1.137, 3.066) times higher. Stroke survivors with moderately severe (IRR: 0.649; 95% CI: 0.490, 0.859) stroke were less likely to have PC visits over 4–12 months post-stroke as compared to those with mild stroke.

**Table 2:** Association of caregiver and patient characteristics with outpatient medical follow-up (primary care) 0–3 months post-stroke

Variable	Reference category	Primary care visits (0 - 3 months)			
		IRR (95% CI)	***P***-value	aIRR^a^ (95% CI)	***P***-value
**Caregiver factors**					
**Age (in years)**		1.013 (1.002, 1.023)	0.017		
**Gender**	Male	0.845 (0.628, 1.136)	0.264		
**Ethnicity**	Non-Chinese	1.195 (0.912, 1.567)	0.197		
**Marital status**	Single	0.917 (0.671, 1.254)	0.589		
**Caregiver relationship**	Spouse		0.443		
**Adult-child**		0.809 (0.594, 1.101)			
**Sibling**		0.641 (0.283, 1.451)			
**Others**		0.943 (0.554, 1.605)			
**Comorbid conditions**	None		0.829		
**1**		0.856 (0.610, 1.202)			
**2**		0.924 (0.578, 1.477)			
**≥3**		1.008 (0.631, 1.611)			
**Co-residing with patient**	No	1.290 (0.815, 2.043)	0.277		
**Caring for multiple care recipients**	No	0.947 (0.724, 1.238)	0.691		
***Revised memory and behavior checklist***					
**Memory problems**		0.937 (0.910, 0.965)	<0.001	0.954 (0.919, 0.990)	0.013
**Depressive behavior problems**		0.959 (0.927, 0.992)	0.015		
**Disruptive behavior problems**		0.981 (0.943, 1.020)	0.333		
***Caregiver burden***					
**Oberst caregiving burden scale**		0.972 (0.960, 0.984)	<0.001	0.976 (0.959, 0.993)	0.005
**Zarit burden interview**		0.994 (0.977, 1.012)	0.523		
***Family conflict***					
**Attitude towards patient**		0.971 (0.943, 0.999)	0.041		
**Attitude towards caregiver**		0.966 (0.938, 0.995)	0.021		
***Social support (instrumental)***					
**FDW for general help**	No	1.599 (1.076, 2.376)	0.020		
**FDW for stroke patient**	No	0.455 (0.265, 0.781)	0.004		
**Social support (perceived)**		1.011 (0.984, 1.039)	0.427		
***Care management strategies***					
**Positive strategies**		0.981 (0.969, 0.993)	0.002		
**Negative strategies**		0.995 (0.966, 1.024)	0.737		
**Patient factors**					
**Age (in years)**		1.001 (0.989, 1.014)	0.827		
**Gender**	Male	0.853 (0.643, 1.130)	0.268		
**Ethnicity**	Non-Chinese	1.094 (0.836, 1.431)	0.515		
**Marital status**	Single	1.254 (0.878, 1.792)	0.214		
**Ward class**	Unsubsidized	1.437 (0.820, 2.516)	0.205		
**CCI**	1 - 3		0.593		
**4 - 6**		1.202 (0.844, 1.711)			
**>= 7**		1.175 (0.749, 1.842)			
**Stroke type**	Non-ischemic	2.451 (1.337, 4.493)	0.004	2.327 (1.110, 4.876)	0.025
**Recurrent stroke**	No	0.827 (0.566, 1.209)	0.327		
**National Institute of Health Scale**	Mild (0-4)		<0.001		
**Moderately severe (5-14)**		0.594 (0.443, 0.797)			
**Severe (15-24)**		0.266 (0.098, 0.716)			
**Modified rankin scale**	No or slight disability (0-2)				
**Moderate or severe disability (3-5)**		0.602 (0.462, 0.784)	<0.001		
**Mini-mental state examination**	No cognitive impairment (24-30)		<0.001		
**Mild cognitive impairment (18-23)**		0.696 (0.503, 0.965)			
**Severe cognitive impairment (1-17)**		0.422 (0.265, 0.671)			
**Discharge to community hospital**	No	0.569 (0.398, 0.813)	0.002		
**Centre for epidemiological studies depression scale**		1.008 (0.985, 1.032)	0.495		

*Abbreviations*: *IRR* incidence rate ratio, *aIRR* adjusted incidence rate ratio, *CI* confidence interval, *FDW* foreign domestic worker

^a^Model adjusted for age, gender, ethnicity and ward class of the patient

**Table 3:** Association of caregiver and patient characteristics with outpatient medical follow-up (primary care) 4–12 months post-stroke

Variable	Reference category	Primary care visits (4 - 12 months)			
		IRR (95% CI)	***P***-value	aIRR^a^ (95% CI)	***P***-value
**Caregiver factors**					
**Age (in years)**		1.005 (0.999, 1.011)	0.127		
**Gender**	Male	0.978 (0.810, 1.180)	0.816		
**Ethnicity**	Non-Chinese	0.981 (0.833, 1.154)	0.816		
**Marital status**	Single	1.050 (0.860, 1.281)	0.633		
**Caregiver relationship**	Spouse		0.115		
**Adult-child**		0.989 (0.823, 1.187)			
**Sibling**		0.606 (0.355, 1.034)			
**Others**		1.248 (0.926, 1.682)			
**Comorbid conditions**	None		0.418		
**1**		1.124 (0.925, 1.365)			
**2**		1.093 (0.830, 1.441)			
**≥3**		0.869 (0.633, 1.194)			
**Co-residing with patient**	No	1.218 (0.925, 1.604)	0.160		
**Caring for multiple care recipients**	No	0.998 (0.847, 1.176)	0.980		
***Revised memory and behavior checklist***					
**Memory problems**		0.988 (0.974, 1.003)	0.108		
**Depressive behavior problems**		0.980 (0.962, 0.999)	0.035		
**Disruptive behavior problems**		0.996 (0.974, 1.019)	0.714		
***Caregiver burden***					
**Oberst caregiving burden scale**		0.989 (0.982, 0.995)	0.001		
**Zarit burden interview**		0.994 (0.983, 1.004)	0.232		
***Family conflict***					
**Attitude towards patient**		0.978 (0.961, 0.995)	0.013		
**Attitude towards caregiver**		0.987 (0.970, 1.006)	0.173		
***Social support (instrumental)***					
**FDW for general help**	No	1.233 (0.988, 1.539)	0.064		
**FDW for stroke patient**	No	0.681 (0.515, 0.901)	0.007		
**Social support (perceived)**		1.000 (0.984, 1.017)	0.987		
***Care management strategies***					
**Positive strategies**		0.996 (0.989, 1.004)	0.332		
**Negative strategies**		0.995 (0.978, 1.013)	0.600		
**Patient factors**					
**Age (in years)**		0.998 (0.991, 1.006)	0.686		
**Gender**	Male	0.828 (0.696, 0.986)	0.034		
**Ethnicity**	Non-Chinese	0.899 (0.764, 1.058)	0.199		
**Marital status**	Single	1.148 (0.928, 1.419)	0.204		
**Ward class**	Unsubsidized	1.137 (0.833, 1.551)	0.418		
**CCI**	1 - 3		0.002		0.023
**4 - 6**		1.526 (1.207, 1.928)		1.655 (1.152, 2.378)	
**>= 7**		1.471 (1.102, 1.963)		1.590 (0.999, 2.527)	
**Stroke type**	Non-ischemic	1.805 (1.304, 2.497)	<0.001	1.867 (1.137, 3.066)	0.014
**Recurrent stroke**	No	0.893 (0.712, 1.120)	0.328		
**National Institute of Health Scale**	Mild (0-4)		<0.001		0.010
**Moderately severe (5-14)**		0.686 (0.575, 0.819)		0.649 (0.490, 0.859)	
**Severe (15-24)**		0.558 (0.360, 0.865)		0.813 (0.414, 1.596)	
**Modified rankin scale**	No or slight disability (0-2)				
**Moderate or severe disability (3-5)**		0.724 (0.616, 0.851)	<0.001		
**Mini-mental state examination**	No cognitive impairment (24-30)		0.003		
**Mild cognitive impairment (18-23)**		1.086 (0.903, 1.307)			
**Severe cognitive impairment (1-17)**		0.679 (0.528, 0.874)			
**Discharge to community hospital**	No	0.766 (0.628, 0.935)	0.009		
**Centre for epidemiological studies depression scale**		1.006 (0.992, 1.021)	0.387		

*Abbreviations*: *IRR* incidence rate ratio, *aIRR* adjusted incidence rate ratio, *CI* confidence interval, *FDW* foreign domestic worker

^a^Model adjusted for age, gender, ethnicity and ward class of the patient

### Outpatient medical (SOC) healthcare utilization

Table 4 depicts the results of the association of caregiver characteristics with the rate of SOC visits across three months post-stroke. None of the caregiver or patient variables entered the final adjusted model of SOC visits over three months post-stroke. The variables that entered the final adjusted model of SOC visits over 4–12 months post-stroke were co-residing caregiver, negative care management strategies, functional status and discharge destination post-stroke. (Please refer to Table 5) Compared to those with no co-residing caregiver, those with co-residing caregivers had almost 1.6 times greater rate of SOC visits over 4–12 months post-stroke (IRR: 1.576; 95% CI: 1.040, 2.389). With every 1 unit increase in negative care management strategy score, the rate ratio of SOC visits increased by a factor of 1.033 (95% CI: 1.005, 1.061). Compared to those with no or mild disability, those with moderate to severe disabilities had 1.564 times greater rate of SOC visits over 4–12 months post-stroke (95% CI: 1.197, 2.043). Being discharged to community hospital after the index stroke was associated with a higher rate of SOC visits in 4–12 months post-stroke.

**Table 4:** Association of caregiver and patient characteristics with outpatient medical follow-up (specialist outpatient care) 0–3 months post-stroke

Variable	Reference category	SOC Visits (0 - 3 months)			
		IRR (95% CI)	***P***-value	aIRR^a^ (95% CI)	***P***-value
**Caregiver factors**					
**Age (in years)**		0.999 (0.992, 1.006)	0.780		
**Gender**	Male	1.187 (0.951, 1.483)	0.130		
**Ethnicity**	Non-Chinese	0.992 (0.827, 1.191)	0.934		
**Marital status**	Single	0.975 (0.784, 1.213)	0.820		
**Caregiver relationship**	Spouse		0.380		
**Adult-child**		0.959 (0.782, 1.175)			
**Sibling**		0.946 (0.588, 1.522)			
**Others**		0.680 (0.441, 1.049)			
**Comorbid conditions**	None		0.857		
**1**		1.021 (0.818, 1.274)			
**2**		0.875 (0.626, 1.223)			
**≥3**		0.955 (0.683, 1.335)			
**Co-residing with patient**	No	1.269 (0.927, 1.736)	0.137		
**Caring for multiple care recipients**	No	0.977 (0.813, 1.175)	0.807		
***Revised memory and behavior checklist***					
**Memory problems**		1.005 (0.990, 1.020)	0.486		
**Depressive behavior problems**		1.018 (1.001, 1.036)	0.040		
**Disruptive behavior problems**		1.004 (0.979, 1.028)	0.773		
***Caregiver burden***					
**Oberst caregiving burden scale**		1.004 (0.997, 1.011)	0.308		
**Zarit burden interview**		0.997 (0.986, 1.009)	0.670		
***Family conflict***					
**Attitude towards patient**		1.011 (0.991, 1.032)	0.295		
**Attitude towards caregiver**		1.013 (0.992, 1.034)	0.233		
***Social support (instrumental)***					
**FDW for general help**	No	1.016 (0.805, 1.282)	0.894		
**FDW for stroke patient**	No	1.085 (0.835, 1.409)	0.544		
**Social support (perceived)**		0.984 (0.966, 1.002)	0.076		
***Care management strategies***					
**Positive strategies**		0.996 (0.988, 1.005)	0.396		
**Negative strategies**		1.008 (0.988, 1.027)	0.443		
**Patient factors**					
**Age (in years)**		0.994 (0.985, 1.002)	0.156		
**Gender**	Male	1.027 (0.851, 1.240)	0.778		
**Ethnicity**	Non-Chinese	0.961 (0.801, 1.154)	0.670		
**Marital status**	Single	1.011 (0.804, 1.271)	0.926		
**Ward class**	Unsubsidized	1.111 (0.787, 1.567)	0.550		
**CCI**	1 - 3		0.086		
**4 - 6**		1.137 (0.891, 1.451)			
**>= 7**		1.387 (1.032, 1.865)			
**Stroke type**	Non-ischemic	0.796 (0.613, 1.034)	0.088		
**Recurrent stroke**	No	1.308 (1.045, 1.637)	0.019		
**National Institute of Health Scale**	Mild (0-4)		0.021		
**Moderately severe (5-14)**		0.779 (0.639, 0.948)			
**Severe (15-24)**		1.151 (0.799, 1.658)			
**Modified rankin scale**	No or slight disability (0-2)				
**Moderate or severe disability (3-5)**		1.081 (0.898, 1.301)	0.411		
**Mini-mental state examination**	No cognitive impairment (24-30)		0.756		
**Mild cognitive impairment (18-23)**		1.034 (0.835, 1.280)			
**Severe cognitive impairment (1-17)**		0.928 (0.721, 1.196)			
**Discharge to community hospital**	No	0.952 (0.771, 1.175)	0.646		
**Centre for epidemiological studies depression scale**		1.010 (0.994, 1.027)	0.208		

*Abbreviations*: *IRR* incidence rate ratio, *aIRR* adjusted incidence rate ratio, *CI* confidence interval, *FDW* foreign domestic worker

^a^Model adjusted for age, gender, ethnicity and ward class of the patient

**Table 5:** Association of caregiver and patient characteristics with outpatient medical follow-up (specialist outpatient care) 4–12 months post-stroke

Variable	Reference category	SOC visits (4 - 12 months)			
		IRR (95% CI)	***P***-value	aIRR^a^ (95% CI)	***P***-value
**Caregiver factors**					
**Age (in years)**		0.997 (0.993, 1.001)	0.176		
**Gender**	Male	0.986 (0.863, 1.127)	0.834		
**Ethnicity**	Non-Chinese	1.355 (1.202, 1.527)	0.000		
**Marital status**	Single	1.007 (0.876, 1.157)	0.927		
**Caregiver relationship**	Spouse		<0.001		
**Adult-child**		1.257 (1.110, 1.423)			
**Sibling**		1.282 (0.974, 1.688)			
**Others**		0.823 (0.632, 1.071)			
**Comorbid conditions**	None		0.966		
**1**		0.983 (0.853, 1.133)			
**2**		1.006 (0.824, 1.227)			
**≥3**		0.950 (0.768, 1.175)			
**Co-residing with patient**	No	1.254 (1.030, 1.527)	0.024	1.576 (1.040, 2.389)	0.032
**Caring for multiple care recipients**	No	0.996 (0.887, 1.118)	0.944		
***Revised memory and behavior checklist***					
**Memory problems**		1.011 (1.002, 1.021)	0.016		
**Depressive behavior problems**		1.013 (1.002, 1.025)	0.018		
**Disruptive behavior problems**		1.017 (1.002, 1.032)	0.024		
***Caregiver burden***					
**Oberst caregiving burden scale**		1.017 (1.013, 1.022)	<0.001		
**Zarit burden interview**		1.016 (1.010, 1.023)	<0.001		
***Family conflict***					
**Attitude towards patient**		0.983 (0.971, 0.995)	0.007		
**Attitude towards caregiver**		0.984 (0.971, 0.996)	0.012		
***Social support (instrumental)***					
**FDW for general help**	No	0.755 (0.660, 0.864)	<0.001		
**FDW for stroke patient**	No	1.414 (1.216, 1.644)	<0.001		
**Social support (perceived)**		0.984 (0.973, 0.995)	0.005		
***Care management strategies***					
**Positive strategies**		1.006 (1.001, 1.011)	0.027		
**Negative strategies**		1.035 (1.024, 1.046)	<0.001	1.033 (1.005, 1.061)	0.021
**Patient factors**					
**Age (in years)**		0.995 (0.990, 1.001)	0.101		
**Gender**	Male	1.083 (0.963, 1.219)	0.183		
**Ethnicity**	Non-Chinese	1.357 (1.204, 1.530)	<0.001		
**Marital status**	Single	0.862 (0.751, 0.990)	0.035		
**Ward class**	Unsubsidized	0.975 (0.794, 1.198)	0.808		
**CCI**	1 - 3		<0.001		
**4 - 6**		1.175 (1.004, 1.375)			
**>= 7**		1.601 (1.330, 1.927)			
**Stroke type**	Non-ischemic	0.904 (0.760, 1.075)	0.253		
**Recurrent stroke**	No	1.175 (1.015, 1.361)	0.030		
**National Institute of Health Scale**	Mild (0-4)		<0.001		
**Moderately severe (5-14)**		1.237 (1.098, 1.392)			
**Severe (15-24)**		1.359 (1.075, 1.718)			
**Modified rankin scale**	No or slight disability (0-2)				
**Moderate or severe disability (3-5)**		1.520 (1.344, 1.719)	<0.001	1.564 (1.197, 2.043)	0.001
**Mini-mental state examination**	No cognitive impairment (24-30)		0.007		
**Mild cognitive impairment (18-23)**		1.078 (0.939, 1.237)			
**Severe cognitive impairment (1-17)**		1.266 (1.092, 1.466)			
**Discharge to community hospital**	No	1.475 (1.307, 1.665)	<0.001	1.362 (1.015, 1.826)	0.039
**Centre for epidemiological studies depression scale**		0.996 (0.985, 1.007)	0.445		

*Abbreviations*: *IRR* incidence rate ratio, *aIRR* adjusted incidence rate ratio, *CI* confidence interval, *FDW* foreign domestic worker

^a^Model adjusted for age, gender, ethnicity and ward class of the patient

## Discussion

We are among the first to describe the role of caregivers in outpatient medical follow-up post-stroke. Adding new knowledge to existing literature, we demonstrated that the association of caregiver factors with outpatient medical visits varied by the type of service consumed (i.e., PC versus SOC) and temporally across the early and late post-stroke period within each service. We found that caregiver factors were significantly associated with PC visits in the early post-stroke period only, with patient factors being significantly associated with early and late post-stroke periods. A possible explanation could be related to the caregivers stepping into the new caregiving role during the early period, which is often reported as a challenging and overwhelming experience [15, 26, 27]. Once caregivers transition into a stable phase with role familiarity, it may be possible that clinical factors solely determined the frequency of PC visits.

We reported a higher mean number of SOC visits than PC visits for both 0–3 months and 4–12 months post-stroke. Contrary to our finding, previous studies have reported stroke survivors to have higher primary care visits as compared to specialist visits over 1 month [28], 3 months [29] and 12 months [5] post-stroke. It may be possible that stroke survivors in our setting continued being treated in SOC clinics without timely transfer to PC clinics. It is well-established that specialist settings encounter high patient load within Singapore, which may be transferred to primary care setting, ensuring the right care in the right setting. This preposition of treating patients in the most appropriate setting at the lowest possible cost and achieving favourable patient outcomes is known as “right-siting” of healthcare services. This term has been commonly used in Singapore since 2004 to describe the principle of transferring patients with stable chronic conditions from SOC clinics to PC clinics [30]. While such “right-siting” programs have been established for patients with chronic kidney disease [31], asthma [32] and diabetes [30] there has been no such program for stroke survivors. Another possibility explaining the difference in our finding with existing literature could be related to differences in patient profiles; specifically, stroke survivors in our study may require specialist care for post-stroke needs. Further exploration of the transition between SOC and PC settings post-stroke is beyond the scope of the current study, which aimed to study the caregiver and patient factors associated with the use of PC and SOC services. However, acknowledging the importance of treating patients in the most appropriate care setting, future research should examine transitions across different outpatient medical care settings to optimize the use of such outpatient services.

While there is unequivocal evidence in both non-stroke [33, 34] and stroke populations [13, 35] of caregiver burden being associated with the increased use of inpatient services, the role of caregiver burden in the use of outpatient clinical services is unexplored and unestablished. Within the stroke population, caregiver burden is reported to be associated with increased hospitalization [13] and increased institutionalization of stroke survivors [35]. Contrary to the established association of caregiver burden with the increased use of inpatient services, we found that caregiver burden was associated with reduced PC visits post-stroke. Our finding is concordant with previous literature on the utilization of non-urgent services being associated with caregiver burden [36]. Considering the role of caregiver burden in post-stroke inpatient and PC services utilization, it may be possible that burdened caregivers do not engage in outpatient medical follow-up in the early period, which may result in subsequent greater hospitalizations, further highlighting the importance of early outpatient medical follow-up.

We found caregiver-reported memory type of behavioral problems were associated with decreased PC visits. One possible explanation could be caregivers perceiving stroke survivors’ memory problems as part of normal ageing and not cues to seek medical care for post-stroke sequelae [37–39]. Alternatively, handling memory-related behavioral issues may additionally strain the caregivers and they may not have the bandwidth or time to seek PC services. A study comparing healthcare utilization in stroke and non-stroke populations in the Canadian setting reported stroke survivors to have significantly more visits to most healthcare professionals than those without stroke. Further, researchers reported that stroke survivors with mood or anxiety disorders were 1.4 times more likely to visit a family physician than those without these disorders [5]. Differences in findings across both studies could be related to the different types of behavioral issues captured in the analyses (i.e., memory vs mood disorders), differences in data collection methods (i.e., objective source of PC visits from claims records in our study versus self-reported PC visits in the comparison study) or differences in the perception of behavioral problems.

Our findings related to patient determinants of PC visits were in concordance with previous literature, specifically patients with multiple chronic conditions being high utilizers of healthcare services post-stroke compared to those without such chronic conditions [10, 31]. In agreement with our results were the results reported by Roth and colleagues [9], with a higher Charlson comorbidity score being associated with higher consumption of health services. Specifically, stroke survivors with higher scores had longer hospitalization stay, more primary care outpatient visits and increased odds of receiving rehabilitation services.

For SOC visits, we found having a co-residing caregiver associated with increased SOC visits in the late post-stroke period, which is in agreement with the findings from a non-stroke population [40]. While there is consistent evidence supporting the presence of a co-residing caregiver being associated with reduced use of inpatient services [9, 41, 42], such evidence is limited for outpatient medical services. A US-based study on community-dwelling, Medicare enrolled, hospitalized patients explored the association of living alone (without a potential caregiver) with readmission within 2 months post-discharge. They reported the odds of early readmission being 1.5 times in those living alone versus those living with someone [41]. Skinner and colleagues reported the co-residing status of adult–child caregivers being associated with reduced hospital length of stay as compared to those with adult–child caregivers living more than 15 min away from the elderly [42]. Within the stroke population, having a co-residing caregiver was reported to be associated with reduced length of hospitalization and fewer ED visits [9]. It is possible that having a co-residing caregiver ensures availability of support or assistance in the community post-stroke, which may aid in a smooth transition to home, prevent unnecessary use of acute healthcare services and facilitate adherence and attendance to scheduled specialist appointments.

Caregivers’ negative care management strategies were associated with increased SOC visits in the late post-stroke period. This could be explained by the caregivers’ perception of SOC settings and specialists. Past studies have reported a differential preference for specialists over regular doctors for specific conditions [43]. Additionally, caregivers may feel more comfortable sharing their problems during a SOC visit as compared to PC visit since the latter tend to be shorter, with about 89% of PC consults for chronic ailments in Singapore lasting between 6 to 10 min [44]. A third possibility could be that stroke survivors using SOC services may have higher care needs, making caregiving challenging, resulting in caregivers adopting more negative care management strategies. Qualitatively exploring the caregiving challenges in caregivers of stroke survivors visiting SOC and PC clinics may help gain a deeper understanding of this reported association. Similar to our findings, other researchers have reported stroke survivors’ functional status playing a significant role in stroke-related costs [45, 46], and healthcare utilization [45, 47].

After establishing the role of caregivers in outpatient medical follow-up post-stroke, we recommend family physicians view caregivers not only as facilitators of care in the community but also as active members of the care team responsible for patient care and as potential clients requiring care and regular assessments. Viewing caregivers as clients during consults includes physicians assessing caregiver needs [48], coordinating required services and ensuring their well-being as part of the routine post-stroke follow-up. This will result in the stroke survivor-caregiver dyadic well-being and potentially ensure the sustainability of caregiving arrangement and continuity of outpatient medical follow-up. Having a caregiver as an active member of the care team can be operationalized by practicing the concept of “inclusive care” which establishes the caregiver role as “*extending the healthcare team in the home environment and representing their care recipients in the clinic environment*” [49].

### Study strengths

We are the first to determine caregiver factors associated with outpatient medical follow-up post-stroke. The study recruitment extending to all the existing tertiary hospitals in Singapore during the recruitment period increases the representativeness of our findings. Furthermore, we had no language related exclusions, which increases the generalizability of our results. We had an objective source for the outcome measure (i.e., the National Claims Database), which increases the accuracy of data capture and limits the possibility of information bias. Moreover, not relying on participant follow-up to capture the outcome over time limits loss to follow-up bias. These, in turn, improve the internal validity of our study.

### Study limitations

Our study has some limitations. Although we can comment on the directionality of association of caregiver factors with outpatient medical follow-up in the late post-stroke period, having captured the determinants before the outcome, we are limited to comment on the directionality of association of caregiver factors and outpatient medical follow-up in the early post-stroke period as both the determinants and outcomes were captured simultaneously. We excluded stroke survivors who died (*N* = 5) during the yearlong follow-up limiting the generalizability of our findings to patients who survive first year post-stroke.

## Conclusion

In conclusion, we reported caregiver factors significantly associated with both PC and SOC visits, establishing caregivers’ role in outpatient medical follow-up post-stroke. While caregiver-reported memory related behavioral problems of a stroke survivor and caregiver burden were significant associated with lower early post-stroke PC visits, co-residing caregiver and negative care management strategies were significantly associated with higher late post-stroke SOC visits. Our results support family-centered care provision by family physicians, viewing the caregivers not only as facilitators of care in the community but also as active members of the care team and as clients requiring care and regular assessments. Caregivers should be integrated into the care teams by practicing inclusive care, which also ensures the extension of the healthcare team in the home environment.

## Supplementary Information

**Additional file 1.**

## Data Availability

The datasets used and analysed during the current study are available from the corresponding author on reasonable request.

## Abbreviations

CCI: Charlson Comorbidity Index
CI: Confidence interval
IRR: Incidence rate ratio
MMSE: Mini-Mental State Examination
mRS: Modified Rankin Scale
NIHSS: National Institute of Health Scale
PC: Primary care
SOC: Specialist outpatient care
