# Supplementary material for: Role of caregiver factors in outpatient medical follow-up post-stroke: observational study in Singapore
Source: BMC Fam Pract. 2021 Apr 14;22:74. doi: 10.1186/s12875-021-01405-z (PMC8048235; doi:10.1186/s12875-021-01405-z)
Supplement: Supplementary file 1 — Additional file 1. [file 12875_2021_1405_MOESM1_ESM.pdf]

Role of caregiver factors in outpatient medical follow-up post-stroke: observational study in Singapore.

Shilpa Tyagi, PhD; Gerald Choon-Huat Koh*, PhD; Nan Luo, PhD; Kelvin Bryan Tan, PhD; Helen Hoenig, MD; David B. Matchar, MD; Joanne Yoong, PhD; Angelique Chan, PhD; Kim En Lee, FRCP; N. Venketasubramanian, FAMS; Edward Menon, FRCP; Kin Ming Chan, M. Med.; Deidre Anne De Silva, FRCP; Philip Yap, MRCP; Boon Yeow Tan, FCFP; Effie Chew, FAMS; Sherry H. Young, MD; Yee Sien Ng, FAMS; Tian Ming Tu, MRCP; Yan Hoon Ang, FRCP; Keng He Kong, FAMS; Rajinder Singh, FRCP; Reshma A. Merchant, FRCP (Edin); Hui Meng Chang, FAMS; Tseng Tsai Yeo, FRACS; Chou Ning, FRACS; Angela Cheong, BN; Yu Li Ng, B.Sc.; Chuen Seng Tan, PhD

Shilpa Tyagi, Saw Swee Hock School of Public Health, National University of Singapore, Singapore. Email: shilpa.tyagi@u.nus.edu

Gerald Choon-Huat Koh, Saw Swee Hock School of Public Health, National University of Singapore, Singapore. Email: gerald_koh@nuhs.edu.sg

Nan Luo, Saw Swee Hock School of Public Health, National University of Singapore, Singapore. Email: ephln@nus.edu.sg

Kelvin Bryan Tan, Policy Research & Economics Office, Ministry of Health, Singapore. Email: kelvin_bryan_tan@moh.gov.sg

Helen Hoenig, Physical Medicine and Rehabilitation Service, Durham VA Medical Centre, USA. Email: Helen.Hoenig@va.gov

David B. Matchar, Program in Health Services and Systems Research, Duke-NUS Medical School, Singapore. Email: David.matchar@duke-nus.edu.sg

Joanne Yoong, Saw Swee Hock School of Public Health, National University of Singapore, Singapore. Email: joanne.yoong@gmail.com

Angelique Chan, Program in Health Services and Systems Research, Duke-NUS Medical School, Singapore. Email: angelique.chan@duke-nus.edu.sg

Kim En Lee, Lee Kim En Neurology Pte Ltd, Singapore. Email: sgstroke@drlee-neuro.com

N. Venketasubramanian, Raffles Neuroscience Centre, Raffles Hospital, Singapore. Email: drnvramani@gmail.com

Edward Menon, St. Andrew's Community Hospital, Singapore. Email: menon@sach.org.sg

Kin Ming Chan, Mount Alvernia Hospital, Singapore. Email: cckkmm@singnet.com.sg

Deidre Anne De Silva, National Neuroscience Institute, Singapore General Hospital campus, Singapore. Email: deidre.a.de.silva@sgh.com.sg

Philip Yap, Geriatric Medicine, Khoo Teck Puat Hospital, Singapore. Email: yap.philip.lk@ktph.com.sg

Boon Yeow Tan, St. Luke's Hospital, Singapore. Email: boon_yeow_tan@nuhs.edu.sg

Effie Chew, Department of Rehabilitation Medicine, National University Hospital, Singapore. Email: effie_chew@nuhs.edu.sg

Sherry H. Young, Department of Rehabilitation Medicine, Changi General Hospital, Singapore. Email: sherry_young@cgh.com.sg

Yee Sien Ng, Department of Rehabilitation Medicine, Singapore General Hospital, Singapore. Email: ng.yee.sien@singhealth.com.sg

Tian Ming Tu, Department of Neurology, National Neuroscience Institute, Neurology, Tan Tock Seng Hospital, Singapore. Email: tu.tian.ming@singhealth.com.sg

Yan Hoon Ang, Geriatric Medicine, Khoo Teck Puat Hospital, Singapore. Email: cfsayh@nus.edu.sg

Keng He Kong, Department of Rehabilitation Medicine, Tan Tock Seng Hospital, Singapore. Email: keng_he_kong@ttsh.com.sg

Rajinder Singh, Department of Neurology, National Neuroscience Institute, Neurology, Tan Tock Seng Hospital, Singapore. Email: rajinder_singh@nni.com.sg

Reshma A. Merchant, Department of Medicine, Yong Loo Lin School of Medicine, National University of Singapore. Email: reshmaa@nuhs.edu.sg

Hui Meng Chang, National Neuroscience Institute, Singapore General Hospital campus, Singapore. Email: chang.hui.meng@sgh.com.sg

Tseng Tsai Yeo, Department of Neurosurgery, National University Hospital, Singapore. Email: cfsytt@nus.edu.sg

Chou Ning, Department of Neurosurgery, National University Hospital, Singapore. Email: ning_chou@nuhs.edu.sg

Angela Cheong, Saw Swee Hock School of Public Health, National University of Singapore, Singapore. Email: angela_cheong@nus.edu.sg

Yu Li Ng, Policy Research & Economics Office, Ministry of Health, Singapore. Email: ng_yu_li@moh.gov.sg

Chuen Seng Tan, Saw Swee Hock School of Public Health, National University of Singapore, Singapore. Email: ephtcs@nus.edu.sg

Description of independent variables

**1.1.Caregiver Variables**
- **Socio-demographic characteristics**: Age (in years), gender (female=1, male=0), ethnicity (Chinese=1, Non-Chinese=0) and marital status (married=1, single, including divorced, widowed, unmarried=0) were collected.<br>- **Caregiver relationship** variable captured the relationship of family caregiver to the stroke survivor and comprised of spousal, sibling, adult-child and others as options. Others comprised of distant relatives and friends.<br>- **Comorbid status** was self-reported by caregivers as any health condition diagnosed since the last interview from a pre-designated list of 21 diseases (e.g. diabetes, hypertension, depression and others). The total sum of reported health conditions was categorised as none, 1, 2 or 3 and more reported comorbid conditions.<br>- **Co-residing with patient** was included as categorical variable with two options (yes=1, no=0).<br>- **Caring for multiple care recipients** was included as categorical variable with two options (yes=1, no=0).<br>- **Patient behavioural problems**: We used the Revised Memory and Behavioural Problem Checklist to record the caregiver reported occurrence of problematic behaviour by stroke survivors, which has been previously used in the stroke survivors. (1-4) Caregivers were asked whether any of the 21 problematic behaviours (e.g. “asking the same question over and over”, “destroying property”, “crying and tearfulness” etc.) have occurred during the previous week. Responses were recorded on a 5-point Likert scale: 0 = never, 1 = not in the past week, 2 = 1

to 2 times per week, 3 = 3 to 6 times per week and 4 = daily or more often. (5) We summated the total score across the three broad domains of memory related, disruptive and depressive behavioural problems.

- **Caregiver burden** was captured using two measures: Oberst Caregiving Burden Scale (for objective burden estimate) and Zarit's Burden Interview (for subjective burden estimate).
    - **Oberst caregiving burden scale**, reported to have good psychometric properties in stroke population caregivers, was used to capture amount of time spend on different caregiving activities by the caregiver, such as, "medical or nursing treatments", "managing finances, bills and forms related to the patient's illness" and others. Total of 15 items were scored on Likert scale from 1=none to 5 = a great amount and total score ranged from 15 to 75. (6)
    - Caregiver's appraisal of caregiving impact was captured by the **Zarit's Burden Interview**, which involved asking caregivers to rate how often they feel several negatively phrased questions related to their caregiving role. (7) Validated previously in Singapore (8), we used the abbreviated 12-item version for current study with total score ranging from 0 to 48. An example of question posed is, "do you feel that because of the time you spend with your relative that you don't have enough time for yourself?"
- **Family conflict** was measured using the family caregiving conflict instrument suggested by Pearlin and colleagues. (9) It comprises of two areas of conflict: attitude towards stroke patient and attitude towards caregiver. Each includes 4 statements, such as, "show enough respect for the patient". Caregiver's response

was captured on 4-point Likert scale ranging from strongly agree to strongly disagree with higher score showing high family caregiving conflict (range: 4 – 16).

- **Social support** was captured in accordance to the guidance given by Pearlin and colleagues, including "instrumental" and "expressive" dimensions. (9) Instrumental social support comprised of the physical network of caregiver, and for current study, we used two categorical variables documenting the presence of a foreign domestic worker for general household chores and specifically for stroke patient care, respectively. Expressive social support was measured using Pearlin's 8-item perceived social support instrument (Cronbach's alpha of 0.87) with responses captured on 4-point Likert scale and total score ranging from 4 to 32.
- **Care management strategies**: We used the revised dementia management strategies scale to capture care management strategies by stroke survivor's caregivers. The 20-item instrument version has been validated in Singapore (10) and records responses to frequency of adopted strategy on 5-point Likert scale: 1=never, 2=seldom, 3=sometimes, 4=often and 5=most of the time. The instrument comprises of two subscales of positive and negative dimensions, with good reported internal consistency in Singapore population (Cronbach's alpha 0.89 and 0.87 respectively). (10) We summated the total score across these two dimensions of positive and negative care management strategies.

1.2.Patient Variables

- **Socio-demographic characteristics**: Age (in years), gender (female=1, male=0), ethnicity (Chinese=1, Non-Chinese=0), marital status (married=1, single, including divorced, widowed, unmarried=0) and ward class (subsidized=1, unsubsidized=0) were collected.

- **Comorbid status**: Charlson Comorbidity Index was used to measure the burden of comorbidity in stroke survivors. Each condition is weighted by severity and a composite score is calculated for each stroke survivor. (11)
- **Index stroke characteristics**: Stroke type was coded as ischemic and non-ischemic, latter including both haemorrhagic and mixed. Stroke severity was measured using the 11-item version of National Institute of Health scale (NIHSS), which captures information on data fields like level of consciousness, horizontal eye movements and visual field testing, facial paresis, motor strength of both upper and lower limbs, ataxia, sensory impairment, dysarthria and deficits in language spoken. Items are scored on ordinal scale (0 to 2 or 0 to 3 or 0 to 5) with total score ranging from 0 to 42. (12) Information was collected on whether or not the index stroke was a recurrent one.
- **Cognitive impairment** was measured using the Mini-Mental state examination (MMSE) which comprised of 11 main questions focusing on dimensions of orientation, immediate recall, attention, delayed recall, language and construction. Scoring each item as 1 for correct response the total score could be upto 30. (13) MMSE has been validated locally in Singapore before. (14) For current study, we categorized the stroke survivors into the following three categories: no cognitive impairment (24-30), mild cognitive impairment (18-23) and severe cognitive impairment (1-17).
- **Functional status** was measured using the modified Rankin scale, a global disability measure commonly used to assess functional status post-stroke. It comprises of 7 levels of functioning: 0 (no symptom at all), 1 (no significant disability despite symptoms), 2 (slight disability, unable to carry out all previous activities), 3 (moderate disability, requiring some help), 4 (moderately severe

disability), 5 (severe disability, bedridden, incontinent and requiring constant nursing care) and 6 (dead). (15)

- **Depression**: To screen the patients for depression the abbreviated 11-item version of the Centre for Epidemiological Studies Depression scale (CES-D) was used. This version was originally validated by Kohout et al in 1993 (16) for use in elderly population with good internal consistency (Cronbach's alpha=0.76) and subsequently used in Singapore by a local study. (17) Each item is in the form of a statement or behaviour and the participant is asked to indicate how often they felt like that in the previous one week, scored on a 4-point Likert scale from 0 being rarely or none of the time, 1 being some or little of the time, 2 being occasionally or a moderate amount of time and 4 being most or all of the time. The total score was in the range of 0 to 33.
- **Discharge destination** was recorded as a binary variable with patients being discharged to a step-down facility or not. Latter included home discharges.

References:

1. Bakas T, Kroenke K, Plue LD, Perkins SM, Williams LS. Outcomes among family caregivers of aphasic versus nonaphasic stroke survivors. Rehabilitation Nursing. 2006;31(1):33-42.
2. Clark AN, Sander AM, Pappadis MR, L Evans G, Chiou-Tan FY. Caregiver characteristics and their relationship to health service utilization in minority patients with first episode stroke. NeuroRehabilitation. 2010;27(1):95-104.
3. Gonzalez C, Bakas T. Factors associated with stroke survivor behaviors as identified by family caregivers. Rehabilitation Nursing. 2013;38(4):202-11.

4. Haley WE, Allen JY, Grant JS, Clay OJ, Perkins M, Roth DL. Problems and benefits reported by stroke family caregivers: results from a prospective epidemiological study. Stroke. 2009;40(6):2129-33.
5. Teri L, Truax P, Logsdon R, Uomoto J, Zarit S, Vitaliano PP. Assessment of behavioral problems in dementia: the revised memory and behavior problems checklist. Psychology and aging. 1992;7(4):622.
6. Bakas T, Austin JK, Jessup SL, Williams LS, Oberst MT. Time and difficulty of tasks provided by family caregivers of stroke survivors. Journal of Neuroscience Nursing. 2004;36(2):95.
7. Bédard M, Molloy DW, Squire L, Dubois S, Lever JA, O'Donnell M. The Zarit Burden Interview: a new short version and screening version. The Gerontologist. 2001;41(5):652-7.
8. Yap P. Validity and reliability of the Zarit Burden Interview in assessing caregiving burden. Ann Acad Med Singapore. 2010;39:758-63.
9. Pearlin LI, Mullan JT, Semple SJ, Skaff MM. Caregiving and the stress process: An overview of concepts and their measures. The Gerontologist. 1990;30(5):583-94.
10. Tan L, Yap P, Ng WY, Luo N. Exploring the use of the Dementia Management Strategies Scale in caregivers of persons with dementia in Singapore. Aging & mental health. 2013;17(8):935-41.
11. Charlson ME, Pompei P, Ales KL, MacKenzie CR. A new method of classifying prognostic comorbidity in longitudinal studies: development and validation. Journal of chronic diseases. 1987;40(5):373-83.
12. Kwon S, Hartzema AG, Duncan PW, Min-Lai S. Disability measures in stroke: relationship among the Barthel Index, the Functional Independence Measure, and the Modified Rankin Scale. Stroke. 2004;35(4):918-23.

13. Folstein MF, Folstein SE, McHugh PR. “Mini-mental state”: a practical method for grading the cognitive state of patients for the clinician. Journal of psychiatric research. 1975;12(3):189-98.
14. Ng T-P, Niti M, Chiam P-C, Kua E-H. Ethnic and educational differences in cognitive test performance on mini-mental state examination in Asians. The American Journal of Geriatric Psychiatry. 2007;15(2):130-9.
15. Banks JL, Marotta CA. Outcomes validity and reliability of the modified Rankin scale: implications for stroke clinical trials: a literature review and synthesis. Stroke. 2007;38(3):1091-6.
16. Kohout FJ, Berkman LF, Evans DA, Cornoni-Huntley J. Two shorter forms of the CES-D depression symptoms index. Journal of aging and health. 1993;5(2):179-93.
17. Chan A, Raman P, Ma S, Malhotra R. Loneliness and all-cause mortality in community-dwelling elderly Singaporeans. Demographic Research. 2015;32:1361-82.
